# Increased Expressions of OX40 and OX40 Ligand in Patients with Primary Immune Thrombocytopenia

**DOI:** 10.1155/2019/6804806

**Published:** 2019-03-03

**Authors:** Dawei Cui, Yan Lv, Xinwang Yuan, Guoxiang Ruan, Yu Zhang, Cuilin Yan, Dandan Xu, Mengen Lv, Yun Mao, Jianping Cao, Jie Jin, Jue Xie

**Affiliations:** ^1^Department of Blood Transfusion, The First Affiliated Hospital, Zhejiang University School of Medicine, Hangzhou 310003, China; ^2^Department of Laboratory Medicine, The First Affiliated Hospital of Zhejiang Chinese Medical University, Hangzhou 310006, China; ^3^Department of Haematology, The First Affiliated Hospital of Zhejiang Chinese Medical University, Hangzhou 310006, China; ^4^Department of Laboratory Medicine, The First Affiliated Hospital, Zhejiang University School of Medicine, Hangzhou 310003, China; ^5^Department of Haematology, The First Affiliated Hospital, Zhejiang University School of Medicine, Hangzhou 310003, China

## Abstract

**Background:**

OX40, which is also known as tumor necrosis factor receptor superfamily member 4 (TNFRSF4), and its ligand (OX40L) play a critical role in the pathogenesis of autoimmune diseases. Immune thrombocytopenia (ITP), a hemorrhagic autoimmune disorder, is characterized by low platelet counts that are predominantly caused by antiplatelet autoantibodies. In this study, we firstly investigated the clinical significance of OX40 and OX40L expression in the pathogenesis of ITP in patients.

**Methods:**

Fifty-four newly diagnosed ITP patients and 24 healthy controls (HCs) were enrolled in this study. The percentage of OX40^+^CD4^+^T cells among CD4^+^T cells was analyzed by flow cytometry, and the expression levels of *OX40* and *OX40L* mRNA were analyzed by quantitative real-time PCR. Plasma soluble OX40L (sOX40L) levels were analyzed by ELISA, and plasma levels of antiplatelet autoantibodies were analyzed by a solid-phase technique.

**Results:**

Compared with HCs, the frequencies of OX40^+^CD4^+^T cells were significantly increased in ITP patients, particularly in patients with positive antiplatelet autoantibodies compared to those with negative antiplatelet autoantibodies. The elevated frequencies of OX40^+^CD4^+^T cells were negatively correlated with low platelet counts in patients with positive antiplatelet autoantibodies. Plasma sOX40L levels in ITP patients were significantly greater than those in HCs and increased in patients with positive antiplatelet autoantibodies compared to those with negative antiplatelet autoantibodies. Plasma sOX40L levels were negatively correlated with low platelet counts in patients with positive antiplatelet autoantibodies. Additionally, the mRNA expression levels of *OX40* and *OX40L* in PBMCs from ITP patients were also notably greater than those from HCs, and the expression levels of *OX40* and *OX40L* were significantly different in ITP patients with positive and negative antiplatelet autoantibodies.

**Conclusion:**

These data indicated that increased expression levels of OX40 and OX40L were involved in the pathogenesis of ITP, and OX40 and OX40L may be valuable therapeutic targets for ITP.

## 1. Introduction

Immune thrombocytopenia (ITP) is a hemorrhagic autoimmune disease characterized by low platelet counts and an increased risk of bleeding [1–3]. Antiplatelet autoantibodies are the most causative agents for ITP, and antiplatelet autoantibodies can enhance platelet destruction and impair platelet generation by megakaryocytes [3–5]. The diagnosis of ITP relies on clinical features and laboratory examinations and excludes other factors involved in thrombocytopenia [4–6]. Typical characteristics of ITP patients are skin petechiae and bleeding in mucosal, gastrointestinal, or intracranial regions [5–7]. The most important diagnostic criterion for ITP is a low peripheral platelet count (below 100 × 10^9^/L) [2, 6, 8]. The incidence of ITP is approximately 1.9~6.4 per 10^5^ children/year and 3.3~3.9 per 10^5^ adults/year, and the morbidity is gradually increasing [6]. The classification of ITP includes primary and secondary ITP: primary ITP is defined by unclear causes and secondary ITP is caused by clear diseases, treatments, and other etiological factors [4, 6, 8].

OX40 (CD134), which is also known as tumor necrosis factor receptor superfamily member 4 (TNFRSF4), is expressed primarily on activated T cells, including CD4 and CD8 cells, and is also expressed at low levels on natural killer cells (NK) and natural killer T cells [9–11]. OX40 is not constitutively expressed on naïve T cells but is induced by antigen (Ag) stimulation and other signals, including CD28 ligation, CD40-CD40L ligation, and IL-2 [11–13]. The cognate ligand of OX40, OX40L (CD134L or CD252), which is also known as tumor necrosis factor superfamily member 4 (TNFSF4), is predominantly expressed on antigen-presenting cells (APCs), such as dendritic cells (DCs), B cells, and macrophages, and is also expressed on several immune cells, including vascular endothelial cells, mast cells, and activated CD4^+^ and CD8^+^ T cells [13–15]. OX40L expression is promoted by Ag stimulation and other factors, including prostaglandin E2, thymic stromal lymphopoietin (TSLP), and IL-18 [13, 16–18]. The interplay between OX40 and OX40L contributes to T cell survival, memory, and differentiation, promotes effector cytokine secretion, and reduces regulatory T cell function [12, 13, 15, 19, 20]. The interactions of the OX40/OX40L axis play a crucial role in the pathogenesis of autoimmune diseases, including systemic lupus erythematosus (SLE), rheumatoid arthritis (RA), allergic asthma, and type 1 diabetes mellitus (T1DM) [19–24]. However, the role of OX40 and OX40L remains unclear in the pathogenesis of ITP.

In this study, we firstly evaluated the expression levels of OX40 and OX40L in primary ITP patients. The frequency of OX40^+^CD4^+^T cells among CD4^+^T cells, the plasma concentration of sOX40L protein, and OX40 and OX40L mRNA expression levels in PBMCs were significantly increased in primary ITP patients compared with healthy controls (HCs), and their expression was involved in low platelet counts and antiplatelet autoantibodies of ITP patients.

## 2. Materials and Methods

### 2.1. Patients

Between March and September 2018, fifty-four primary ITP patients were enrolled from the Department of Hematology of the First Affiliated Hospital, Zhejiang University School of Medicine, and the First Affiliated Hospital, Zhejiang Chinese Medical University. Twenty-four sex- and age-matched healthy controls (HCs) were recruited from the Healthy Management Center of the First Affiliated Hospital, Zhejiang University School of Medicine. All ITP patients were diagnosed according to consensus guidelines, and the patients and HCs who had cardiovascular disease, diabetes, pregnancy, obesity, infections, or connective tissue diseases were excluded from our study [3]. The median age of patients at the onset of primary ITP and the median age of the HCs were 37 (range, 19-66) and 37 years old (range, 20-68), respectively. The ITP patients had a median platelet count of 43 × 10^9^/L ranging from 6 to 77 × 10^9^/L. Furthermore, the platelet counts in the healthy individuals ranged from 171 to 318 × 10^9^/L with a median count of 247 × 10^9^/L. The main characteristics of these cases are shown in Table 1. This study was approved by the Institutional Research Ethics Committee of the First Affiliated Hospital of Zhejiang University, and informed consent was obtained from all patients and control subjects that were included in this study.

### 2.2. Plasma and Peripheral Blood Mononuclear Cell Isolation

Human fresh peripheral whole blood specimens were obtained from ITP patients and HCs. Plasma was isolated by centrifugation at 1800 ×g for 10 min and was stored at -80°C until use. Then, the remaining blood components were diluted with an equal volume of PBS for the following isolation of peripheral blood mononuclear cells (PBMCs) by density gradient centrifugation with Ficoll-Hypaque solution (CL5020, CEDARLANE, the Netherlands).

### 2.3. Detection of Antiplatelet Autoantibodies

For the ITP patients and HCs, antiplatelet autoantibodies were analyzed according to the platelet crossmatch protocol (MASPAT kit; K1360; Sanquin, Amsterdam, the Netherlands) described in a previous report [5]. Briefly, a monolayer of donor platelets was immobilized by centrifugation onto the surfaces of microplate wells coated with a platelet-specific mouse monoclonal antibody. Patient plasma was incubated in the appropriate wells. After incubation, unbound plasma components were removed by washing three times. Platelet-bound antibodies were analyzed by adding mouse monoclonal anti-human IgG- and human IgG-sensitized erythrocytes. A positive reaction was indicated by the adherence of red cells containing the MASPAT indicator throughout the well surface, whereas a negative reaction was indicated by discrete pellets of red cells containing the MASPAT indicator in the middle of the well. The experiment was conducted in triplicate.

### 2.4. Detection of Plasma sOX40L

Plasma levels of sOX40L were analyzed by a Quantikine Human OX40 Ligand ELISA Kit (R&D Systems, Minneapolis, MN, USA) in accordance with the manufacturer's protocol.

### 2.5. Flow Cytometry Analysis

Human PBMCs were transferred to sterile tubes and washed twice with phosphate-buffered saline (PBS) and then immunostained with APC-conjugated anti-human OX40 and FITC-conjugated anti-human CD4 (BioLegend, San Diego, CA). Isotype-matched antibody controls were used in this study. All staining procedures were performed according to the manufacturers' protocols, and the stained cells were analyzed by a BD FACSCalibur flow cytometer and CellQuest software (Becton Dickinson, Sparks, MD, USA).

### 2.6. Total RNA Extraction and Quantitative Real-Time PCR

To analyze the mRNA expression of *OX40* and *OX40L* in human PBMCs from patients, total RNA was extracted by the TRIzol reagent (Invitrogen, Carlsbad, CA, USA) according to the manufacturer's instructions. Next, cDNA was synthesized by a PrimeScript™ II 1st Strand cDNA Synthesis Kit (TaKaRa, Dalian, China) according to the manufacturer's protocol.

Real-time PCR was performed using TaKaRa SYBR Supermix (TaKaRa, Dalian, China) on an ABI 7500 analysis system (Applied Biosystems, Foster City, CA, USA). The amplification conditions were as follows: predenaturation (95°C for 30 s), 40 cycles of denaturation (95°C for 30 s), and annealing and extension (60°C for 34 s). The primers were designed and synthesized with the following sequences: sense, 5′-ACAACGACGTGGTCAGCTCCAA-3′, and antisense, 5′-CAGCGGCAGACTGTGTCCTGT-3′ (*OX40*); sense, 5′-CCTACATCTGCCTGCACTTCTC-3′, and antisense, 5′-TGATGACTGAGTTGTTCTGCACC-3′ (*OX40L*); and sense, 5′-GTCTCCTCTGACTTCAACAGCG-3′, and antisense, 5′-ACCACCCTGTTGCTGTAGCCAA-3′ (*GAPDH*). The relative expression levels of the target genes were calculated by the comparative Ct method presented as 2^-ΔΔCt^. The experiments were conducted in triplicate.

### 2.7. Statistical Analysis

The data were analyzed using GraphPad Prism 5.0 software and SPSS 16.0 statistics software, and the descriptive data are presented as medians (ranges) for continuous variables. The data of differential expression between the ITP and control groups from the qRT-PCR assay were analyzed by unpaired Student's *t*-tests, and the results are presented as the mean ± SEM. Correlations between variables were determined using Spearman's correlation coefficient. A *p* value < 0.05 was considered to be statistically significant.

## 3. Results

### 3.1. Detection of Antiplatelet Autoantibodies in ITP Patients

**Figure 1:**
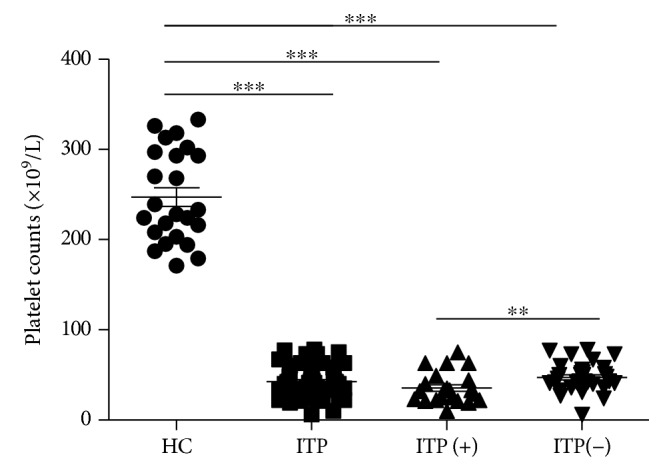
Detection of antiplatelet autoantibodies in ITP patients. Twenty-two (41%) of 54 patients with primary ITP were positive for antiplatelet autoantibodies detected by ELISA. The peripheral platelet counts in patients with positive antiplatelet autoantibodies (ITP(+)) and negative antiplatelet autoantibodies (ITP(-)) and in HCs. ^∗∗∗^*p* < 0.001; ^∗∗^*p* < 0.01; ^∗^*p* < 0.05.

In this study, 22 (41%) of 54 patients with primary ITP were positive for antiplatelet autoantibodies (Table 1). The platelet counts in patients with positive antiplatelet autoantibodies were significantly less than those in patients with negative antiplatelet autoantibodies and in the HCs (Figure 1).

### 3.2. Expression of OX40 on CD4^+^ T Cells in ITP Patients

**Figure 2:**
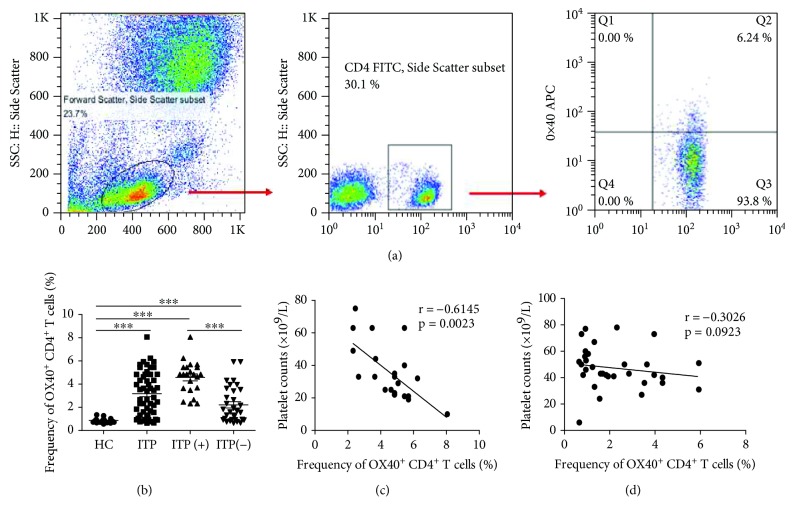
The expression of OX40 on CD4^+^ T cells in ITP patients. Peripheral blood mononuclear cells (PBMCs) from 54 patients with ITP and 24 healthy controls (HCs) were isolated and stained with labeled antibodies and analyzed by flow cytometry, as described in Section 2. (a) The cells were gated initially on lymphocytes and then on CD4^+^ T cells. (b) The frequency of OX40^+^CD4^+^ T cells among the total CD4^+^ T cells from HCs and ITP patients. (c) The relationship between the frequency of OX40^+^CD4^+^ T cells and peripheral platelet counts in ITP patients with positive antiplatelet autoantibodies. (d) The relationship between the frequency of OX40^+^CD4^+^ T cells and peripheral platelet counts in ITP patients with negative antiplatelet autoantibodies. ITP(+): ITP patients with positive antiplatelet autoantibodies; ITP(-): ITP patients with negative antiplatelet autoantibodies. ^∗∗∗^*p* < 0.001; ^∗∗^*p* < 0.01; ^∗^*p* < 0.05.

To investigate the potential role of OX40^+^CD4^+^ T cells in ITP patients, the frequency of circulating OX40^+^CD4^+^ T cells among CD4^+^ T cells was analyzed by flow cytometry (Figure 2(a)). We analyzed OX40 expression gated on CD4^+^ T cells from human PBMCs to delineate circulating OX40^+^CD4^+^ T cells in peripheral blood from ITP patients and HCs. The frequencies of circulating OX40^+^CD4^+^ T cells among CD4^+^ T cells in ITP patients were significantly increased compared to those in HCs; in particular, the frequencies of circulating OX40^+^CD4^+^ T cells in ITP patients with positive antiplatelet autoantibodies were notably greater than those in ITP patients with negative antiplatelet autoantibodies (Figure 2(b)). Interestingly, a significant negative correlation was observed between the frequency of OX40^+^CD4^+^ T cells and peripheral platelet counts in ITP patients with positive antiplatelet autoantibodies (Figure 2(c)), but this correlation was not observed in ITP patients with negative antiplatelet autoantibodies (Figure 2(d)).

### 3.3. Plasma Concentrations of Soluble OX40L in Patients

**Figure 3:**
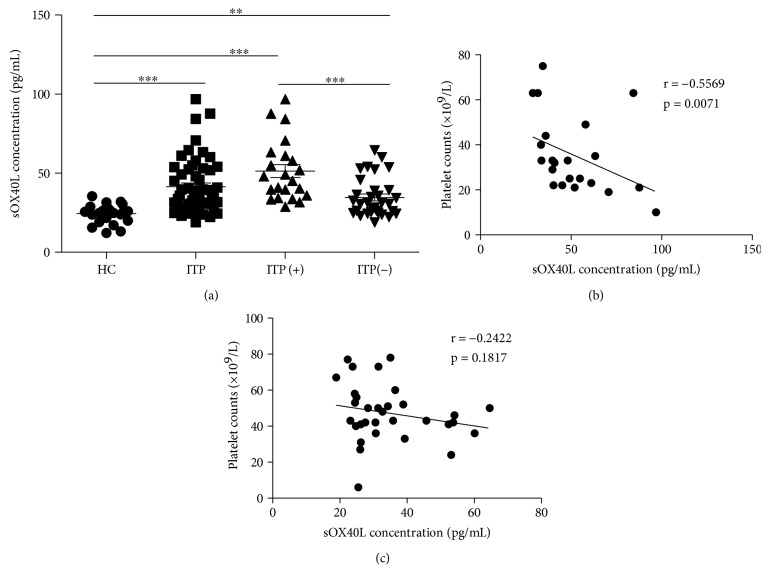
Plasma concentrations of soluble OX40L in patients. (a) Concentrations of plasma sOX40L in HCs and in ITP patients with negative and positive antiplatelet autoantibodies. (b) The relationship between the levels of plasma sOX40L and peripheral platelet counts in ITP patients with positive antiplatelet autoantibodies. (c) The relationship between the levels of plasma sOX40L and peripheral platelet counts in ITP patients with negative antiplatelet autoantibodies. ITP(+): ITP patients with positive antiplatelet autoantibodies; ITP(-): ITP patients with negative antiplatelet autoantibodies. ^∗∗∗^*p* < 0.001; ^∗∗^*p* < 0.01; ^∗^*p* < 0.05.

To analyze the potential role of the OX40 ligand in ITP patients, plasma soluble OX40L (sOX40L) levels were measured by ELISA. The results showed that the concentrations of the plasma sOX40L protein in ITP patients were significantly greater than those in the HCs, and further analysis indicated that there was an obvious difference between ITP patients with positive and negative antiplatelet autoantibodies (Figure 2(a)). Moreover, the levels of the sOX40L protein were obviously correlated with platelet counts in ITP patients with positive antiplatelet autoantibodies (Figure 3(b)), but these parameters were not significantly related in ITP patients with negative antiplatelet autoantibodies (Figure 3(c)).

### 3.4. Expression of *OX40* and *OX40L* mRNA in PBMCs of Patients

**Figure 4:**
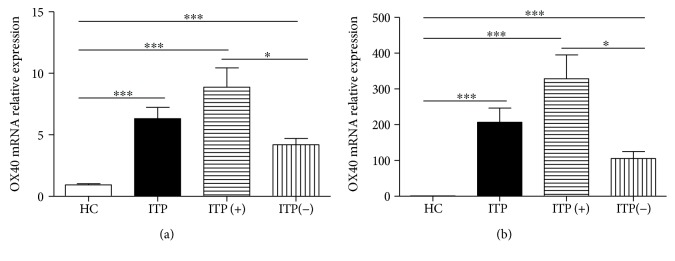
The expression of *OX40* and *OX40L* mRNA in PBMCs of patients. (a) The expression levels of *OX40* mRNA in PBMCs from 10 ITP patients with positive antiplatelet autoantibodies, 12 ITP patients with negative antiplatelet autoantibodies, and 12 HCs. (b) The expression levels of *OX40L* mRNA in PBMCs from 10 ITP patients with positive antiplatelet autoantibodies, 12 ITP patients with negative antiplatelet autoantibodies, and 12 HCs. ITP(+): ITP patients with positive antiplatelet autoantibodies; ITP(-): ITP patients with negative antiplatelet autoantibodies. ^∗∗∗^*p* < 0.001; ^∗∗^*p* < 0.01; ^∗^*p* < 0.05.

To further explore the expression of OX40 and OX40L in ITP patients, the expression levels of *OX40* and *OX40L* mRNA in PBMCs of ITP patients and HCs were comparatively analyzed by quantitative real-time PCR. The results showed remarkably elevated expression levels of *OX40* and *OX40L* mRNA in ITP patients compared with the HCs; in particular, a noticeable difference was observed between ITP patients with positive and negative antiplatelet autoantibodies (Figures 4(a) and 4(b)).

## 4. Discussion

ITP is a complex immune-mediated autoimmune disease characterized by a transient or persistent decreased platelet count due to the increased destruction and decreased production of platelets, which is predominantly caused by the antiplatelet autoantibody. Therefore, the antiplatelet autoantibody is a major causative factor for the pathogenetic mechanism of ITP [1–4]. Antiplatelet autoantibodies mediate platelet destruction by binding to platelet membrane glycoproteins (GPs), including GPIIb/IIIa, GPIb/IX, and GPIV, and impair or inhibit platelet production by megakaryocytes by specifically recognizing platelet antigens located on megakaryocytes (such as GPIb and GPIIb/IIIa) [3–7]. In our study, 40% of ITP patients had positive antiplatelet autoantibodies as well as low platelet counts in comparison with patients with negative antiplatelet autoantibodies, indicating that antiplatelet autoantibodies might aggravate platelet destruction and impair platelet production that causes reduced peripheral platelet counts. Accumulating evidence has shown that some autoimmune diseases can be effectively relieved or even cured by decreasing autoantibody levels using drugs and/or plasma exchange treatments [25–27]. Therefore, we speculated that the disease severity of ITP in patients, specifically in those patients with positive antiplatelet autoantibodies, could be effectively improved or even cured by reducing antiplatelet autoantibody levels by some drug treatments and/or plasma exchange strategies.

Increasing evidence has demonstrated that the OX40-OX40L axis plays an important role in the pathogenesis of autoimmune diseases and is involved in autoantibody production [10–15]. For example, an increased expression level of OX40 on CD4^+^ T cells is closely correlated with disease activity and lupus nephritis, and serum levels of OX40L are positively correlated with anti­dsDNA levels in SLE patients [13, 21, 28]. Plasma levels of soluble OX40L (sOX40L) and the percentages of OX40^+^CD4^+^ T cells in PBMCs have been shown to be increased in early RA patients, and sOX40L levels have been shown to be strongly correlated with the levels of anticitrullinated protein antibodies (ACPAs) and IgM-rheumatoid factor (IgM-RF) [29]. Recent studies have indicated that OX40L expression on APCs can promote follicular helper T (Tfh) cell responses, which further exacerbate autoantibody production [17, 19, 21, 30]. Therefore, the OX40-OX40L axis likely contributes to the generation of autoantibodies in many autoimmune diseases.

However, OX40 and OX40L expression remains completely unclear in ITP patients. In this study, we showed, for the first time, that the percentage of OX40^+^CD4^+^ T cells among CD4^+^ T cells was significantly increased in patients with primary ITP compared with the HCs. Plasma sOX40L levels were significantly increased in patients with primary ITP compared to the HCs. Therefore, these findings indicate the important role of OX40 and sOX40L proteins in the pathogenesis of ITP in patients. Furthermore, OX40 expression levels on CD4^+^ T cells and sOX40L levels in ITP patients with positive antiplatelet autoantibodies were notably greater than those in ITP patients with negative autoantibodies. Interestingly, a negative correlation was observed between OX40^+^CD4^+^ T cell frequencies and low platelet counts, and similar results were observed between sOX40L levels and low platelet counts in ITP patients. These results implied that increased OX40 (OX40L) expression levels possibly contributed to the production of antiplatelet autoantibodies that reduced peripheral platelet counts. Therefore, the OX40-OX40L axis possibly provides an amplification loop for the generation of antiplatelet autoantibodies in ITP patients. Furthermore, recent evidence has suggested that blocking the OX40-OX40L axis may be an effective strategy for ameliorating autoimmune diseases, including SLE, RA, colitis, and type 1 diabetes mellitus [13, 31–34]. Therefore, these results imply that the blockade of OX40-OX40L could be a potential therapeutic strategy for ITP.

Increasing studies have shown that upregulated *OX40* mRNA expression in active CD4^+^ T cells and downregulated *OX40* mRNA expression in Treg cells play a critical role in T1DM [35]. *OX40* mRNA expression has been shown to be upregulated in CD4^+^ T cells from patients with active SLE [36]. Moreover, *OX40* and *OX40L* mRNA expression levels are increased in the spleen, lymph node, and nervous tissue of experimental allergic neuritis (EAN) rats, but not in peripheral blood [13]. The deficiency in functional Roquin proteins increases the *OX40* mRNA expression level and Tfh cell differentiation and causes lupus-like autoimmune disease in mice [30]. In this study, our results indicated that the expression levels of OX40 and OX40L mRNA in PBMCs from ITP patients were significantly increased compared to those in PBMCs from the HCs, and the expression levels of *OX40* and *OX40L* mRNA were notably different between ITP patients with positive and negative antiplatelet autoantibodies. These findings further implied that upregulated *OX40* and *OX40L* mRNA expression might play a crucial role in the pathogenesis of ITP.

## 5. Conclusion

In conclusion, our results showed aberrant expression of OX40 and OX40L in peripheral blood from primary ITP patients, and these findings were indicative of the important roles of OX40 and OX40L in the pathogenesis of ITP in patients. The OX40-OX40L axis likely affects the production of antiplatelet autoantibodies that further decrease platelet counts by impairing and/or inhibiting platelet production. Therefore, the present study indicated that the OX40-OX40L axis may be a potential immune therapeutic target for ITP patients in the future. The investigation of a large number of ITP patients and basic experiments should be conducted to determine the roles of the OX40-OX40L axis in the pathogenesis of ITP in the future.

## Figures and Tables

**Table 1:** The clinical characteristics of primary ITP patients and HCs.

Clinical features	ITP	HCs
Number	54	24
Gender (M/F)	15/39	7/17
Age (range)	37 (19-66)	37 (20-68)
Antiplatelet autoantibody (-/+)	32/22	24/0
Platelet count (range) (×10^9^/L)	43 (6-77)	247 (171-318)

Note: M/F: male/female; ITP: immune thrombocytopenia; HCs: healthy controls.

## Data Availability

All data generated or analyzed in this study are included in the present article.
